# Application of neck ultrasound in the diagnosis of sarcoidosis

**DOI:** 10.1186/s12890-021-01769-z

**Published:** 2021-12-15

**Authors:** Mengjun Shen, Ying Zhou, Weiqing Gu, Chengsheng Yin, Yin Wang, Yuan Zhang

**Affiliations:** 1 Department of Ultrasound, Shanghai Pulmonary Hospital, School of Medicine, Tongji University, 507 Zheng Min Road, Shanghai, 200433 China; 2 Department of Respiratory Medicine, Shanghai Pulmonary Hospital, School of Medicine, Tongji University, 507 Zheng Min Road, Shanghai, 200433 China

**Keywords:** Sarcoidosis, Cervical lymphadenopathy, Ultrasound, CEUS, Tuberculosis

## Abstract

**Objective:**

To explore the significance of neck ultrasound (NUS) combined with contrast-enhanced ultrasound (CEUS) in the diagnosis of sarcoidosis.

**Methods:**

88 patients with evidence of intrathoracic lymphadenopathy and suspected sarcoidosis with enlarged cervical lymph nodes underwent NUS, CEUS, fine-needle aspiration and core needle biopsy when technically feasible were retrospectively analyzed in this study. Seven characteristics such as enhanced mode (EM), resolution time, color Doppler flow imaging (CDFI), fading time, peaking state-uniformity, strengthen the area and symmetry were considered to perform the logistic regression model.

**Results:**

Of 88 patients included in this study, sarcoidosis was accounted in 20 cases, tuberculosis in 23 cases, malignancy in 22 cases and inflammatory lymph node in 23 cases. There were statistically significant differences in symmetry, lymphatic hilum, homogeneity, CDFI pattern and elasticity score between the sarcoidosis and non-sarcoidosis groups via NUS. Similarly, we also acknowledged a statistically significant differences in EM, homogeneity, presence or absence of necrosis between the sarcoidosis and non-sarcoidosis groups via CEUS to further group the non-sarcoidosis into tuberculosis, malignancy or inflammatory disorder. The percentage correction of prediction was 90% (18/20).

**Conclusion:**

NUS combined with CEUS has characteristic features in sarcoidosis with cervical lymph node involvement, which is helpful for its diagnosis and differential diagnosis. The binary classification model of NUS combined with CEUS features can help differentiate sarcoidosis from non-sarcoidosis groups.

## Key point

- The NUS combined with CEUS is very essential for diagnosis of sarcoidosis from other granulomatous non-sarcoidosis lesions non-invasively.

## Background

Sarcoidosis is a systemic disease of unknown etiology, with the pathological feature of non-caseous epithelioid granulomas. In addition to the lung and intrathoracic lymph nodes, many organs including cervical lymph node could be involved [1, 2]. Sarcoidosis is a diagnosis of exclusion because of its similarity in the clinical presentations to other lung diseases (such as tuberculosis, fungal infections, lung cancer, and cryptogenic organizing pneumonia) [3]. The confirmatory diagnosis of sarcoidosis is established only when clinic-radiographic findings are supported by histological evidence of non-caseating granulomatous inflammation with other causes of granulomas and local reactions have been reasonably excluded [2, 3]. China has a high prevalence of TB, which causes a significant challenge in differentiating sarcoidosis from other granulomatous lung diseases especially in cases of smear-negative for tuberculosis [4, 5].

Although great efforts have been devoted to establish methodologies that can differentiate sarcoidosis from other lung diseases [4–6], the diagnosis of sarcoidosis still requires histological confirmation to detect the presence of granulomatous inflammation and also to exclude other potential causes of granulomatous inflammation simultaneously [7, 8]. 10.8% of patients with sarcoidosis are reported to have enlarged cervical lymphadenopathy on ultrasound. And previous studies reported that NUS and FNA of cervical lymph nodes is a potential modality that can be used for the diagnosis of sarcoidosis patients [9–12].

Contrast-enhanced ultrasound (CEUS) is a new technique used for real-time assessment of tissue perfusion. It has been widely used to evaluate liver, kidney, pancreas, spleen, ovarian, thyroid, breast, prostate and even lung lesions in the current clinical practice [13–15]. It has been widely performed in identification of sentinel lymph node in breast cancer patients [16–19].

CEUS helps radiologists investigate and characterize focal lesions. Its contrast pool, sensitivity and specificity in diagnosing liver lesions are similar to contrast CT on the contrary CT is more costly and involves higher levels of ionizing radiation exposure. Recently, CEUS has often been used to identify benign and malignant superficial lymph nodes [20–22].

Previous studies have shown that CEUS has a higher sensitivity in identifying small blood vessel reductions or areas of multiple blood vessels that cannot be detected by Doppler technology leading to a better evaluation of LNS classification [23].

Compared with CT and MRI, gray-scale ultrasound combined with CEUS can assess the shape, margins, internal structure, and vascularization of superficial lymph nodes [24]. It improves the accuracy of ultrasound for diagnosis of superficial lymph nodes disorders.

The main limitation of CEUS is the practice mode, because the interpretation of ultrasound images depends on technical skills and the experience of the radiologist. In addition, CEUS is not suitable for patients who have allergic reactions to contrast agents and pregnant women.

Therefore, this study intends to retrospectively analyze the CEUS qualitative diagnosis data of several groups of diseases involving cervical lymph nodes accounting factors such as the enhanced mode of ultrasound contrast agent, degree of enhancement, homogeneity, presence or absence of necrosis, arrival time, peak time, wash-out time, and so on. We aim to explore the characteristics that guide the diagnosis and differential diagnosis of sarcoidosis and provide a new non-invasive diagnostic methodology for sarcoidosis involving the cervical region.

## Methods

### Study design

This was a retrospective observational study. The study protocols were approved by the Institutional Review Boards of Shanghai Pulmonary Hospital (Approval No K18-144).

### Patients and diagnostic criteria

Altogether 88 patients with tissue proven sarcoidosis, tuberculosis (TB), lung cancer or non-specific inflammation were evaluated at the Shanghai Pulmonary Hospital between September 2018 and December 2019 were recruited for this study. All these patients manifested cervical lymphadenopathy and pathological diagnosis of their lymph node were consistent with their clinical diagnosis. The diagnosis of sarcoidosis was established based on the presence of clinical symptoms, radiological features compatible with sarcoidosis, and biopsy evidence of noncaseating epithelioid cell granulomas with other known causes of granulomatosis excluded.

### Conventional US (B-mode and doppler) NUS examination

All of conventional ultrasound scans and CEUS examinations were performed by two senior clinical ultrasonographers with more than 3 years of experience in neck ultrasound (SMJ and WY). Conventional ultrasound scans were performed by a 6–15 MHZ probe (LOGIQ E9, GE, Wauwatosa, WI). A routine ultrasonography on the target lesion was performed by adjusting the focus and depth of the lesion, then selecting the best acoustic window to fully display the lesion boundary and finally evaluating the symmetry of the lymph nodes (unilateral or bilateral), number (1 or > 1), longitudinal meridian (≥ 10 mm or < 10 mm), aspect ratio (< 2 or ≥ 2), lymphatic hilum (present or absent), border (clear or unclear), echo (hypoechoic or hyperechoic), homogeneity (uniform or non-uniform), calcification (present or absent), and fusion (present or absent). Color Doppler flow imaging (CDFI) and elastic imaging were performed after gray-scale ultrasound. The CDFI pattern of lymph nodes can be divided into four types: portal, dendritic, annular and others. The elasticity scoring was performed based on the elastography 5 points proposed by Itoh et al. [25]. The cases were divided into two groups according to elasticity score (≥ 3 or < 3). As shown in Table 1.

**Table.1:** Elasticity scoring system

Score	Performance
0	The focus area is mainly cystic components, showing red, blue and green alternately
1	The lesion area and the surrounding tissue are uniformly green
2	The focus area is mainly green (green area > 90%)
3	The focus area is messy blue-green or mainly blue (blue area is 50–90%)
4	The lesion area is almost blue covered (blue area > 90%)

### Contrast-enhanced ultrasound (CEUS) examination

After conventional ultrasound, the largest diameter nodule was selected before switching to CEUS mode. CEUS was performed by a 3–9 MHZ probe (LOGIQ E9, GE, Wauwatosa, WI), and using a low mechanical index (MI < 0.13). The contrast agent (SonoVue, Bracco SpA, Milan, Italy) was injected intravenously through the anterior cubital vein with a dose of 1.5 ml followed by bolus irrigation with 5 ml normal saline. The timer was started while injecting the contrast agent, followed by 3 min continuous observation and recording of data. It was also made sure that the image and condition settings remained unchanged during the entire imaging process. We observed and recorded the following indicators: the enhanced mode of ultrasound contrast agent (centripetal, centrifugal, polycentric, or annular), degree of enhancement (low, equal or high), homogeneity (uniform or non-uniform), necrosis (present or absent), and arrival time of contrast agent, peak time, and wash-out time.

### Fine-needle aspiration (FNA) and core needle biopsy (CNB)

Abnormal lymph nodes were defined based on a short axis diameter of ≥ 6 mm. When technically feasible, FNA of lymph nodes was performed by a trained physician. If suitable, a core needle biopsy (CNB) was performed on the lymph node using a 18G*10 cm biopsy needle (Duo smart, Gallini s.r.l, Italy). On site cytology was not performed. The presence of noncaseating granulomas with negative stains for mycobacteria and fungi was considered diagnostic of sarcoidosis.

### Statistical analyses

All data were analyzed using the statistical analysis software SPSS 20.0. Continuous variables were presented as mean ± standard deviation (SD) and were compared using t-test. Chi-square test was used for analysing the categorical variables. Independent-samples t-test assuming equal variance and ANOVA were performed with *p* < 0.05 indicated statistically significant. Two classification analysis was performed by R software.

## Results

### General clinical data

Eighty-eight patients were included in this study with 20 cases of sarcoidosis, 23 cases of tuberculosis, 22 cases of malignancy and inflammatory lymph nodes in 23 cases. There was no significant difference in age and sex between the sarcoidosis group and the tuberculosis group (*p* > 0.05) as shown in Table 2. Table 3 reflects the characteristics of our patients with sarcoidosis and cervical lymphadenopathy based on FNA and CNB.

**Table 2:** Demographic information of subjects recruited

Category	Sarcoidosis group	Control subjects		
		Tuberculosis group	Malignancy group	Inflammatory group
Total subjects, n	20	23	22	23
Age, years	47.1 ± 12.7	44.1 ± 15.0	65.6 ± 10.0	57.5 ± 6.2
Gender				
Male	9 (45.0%)	9 (39.1%)	17 (77.3%)	13 (56.5%)
Female	11 (55.0%)	14 (60.9%)	5 (22.7%)	10 (43.5%)
CXR stage 0/I/II/III/IV ^§^	0/4/18/0/0	N/A	N/A	N/A
Biopsy (cases/needles)				
NUS FNA	20/20	23/23	22/22	23/23
NUS CNB	17/20	23/23	22/22	20/23
EBUS-TBNA/EBB/TBLB	19/19	18/18	20/20	20/20
Surgical biopsy	3/3	0/0	2/2	0/0

CXR = chest X-ray; N/A = not applicable; CXR stage: 0 = no adenopathy, no lung infiltrates; stage I = hilar & mediastinal adenopathy only; stage II = hilar & mediastinal adenopathy plus lung infiltrates; stage III = lung infiltrates only; stage IV = pulmonary fibrosis. EBUS-TBNA = endobronchialultrasound-guidedtransbronchialneedleaspiration, EBB = Endobronchialbiopsy; TBLB = transbronchiallungbiopsy

**Table.3:** Characteristics of patients with sarcoidosis and cervical lymphadenopathy

No	Size of cervical LN^a^	Side of cervical LN	Thoracic LN	FNA result	CNB result
1	18	Right	Yes	Adequate but nondiagnostic	Diagnostic
2	9	Bilateral	Yes	Adequate but nondiagnostic	Diagnostic
3	14	Bilateral	Yes	Adequate but nondiagnostic	Diagnostic
4	18	Bilateral	Yes	Adequate but nondiagnostic	Diagnostic
5	13	Bilateral	Yes	Adequate but nondiagnostic	Diagnostic
6	8	Bilateral	Yes	Adequate but nondiagnostic	Diagnostic
7	8	Bilateral	Yes	Adequate but nondiagnostic	Diagnostic
8	11	Bilateral	Yes	Adequate but nondiagnostic	Diagnostic
9^#^	7	Right	Yes	Adequate but nondiagnostic	Not done
10	10	Bilateral	Yes	Adequate but nondiagnostic	Diagnostic
11	13	Bilateral	Yes	Diagnostic	Diagnostic
12^#^	7	Bilateral	Yes	Adequate but nondiagnostic	Not done
13	11	Bilateral	Yes	Adequate but nondiagnostic	Diagnostic
14	8	Bilateral	Yes	Adequate but nondiagnostic	Diagnostic
15	9	Bilateral	Yes	Adequate but nondiagnostic	Diagnostic
16	12	Bilateral	Yes	Adequate but nondiagnostic	Diagnostic
17^#^	15	Bilateral	Yes	Adequate but nondiagnostic	Not done
18	8	Bilateral	Yes	Adequate but nondiagnostic	Diagnostic
19	17	Bilateral	Yes	Adequate but nondiagnostic	Diagnostic
20	14	Bilateral	Yes	Adequate but nondiagnostic	Diagnostic

^a^Diameter of largest lymph node on ultrasound was in millimeter. # Case 9 was dianosed by EBUS-TBNA and EBB. Case 12 and 17 were underwent mediastinal biopsy. CNB, core needle biopsy; FNA, fine-needle aspiration; LN, lymph node

### Neck ultrasound (NUS) features

Table 4 shows significant differences in symmetry, lymphatic hilum, homogeneity, CDFI pattern and elasticity score between the sarcoidosis and non-sarcoidosis groups statistically (*p* < 0.05). Furthermore, specific to sarcoidosis and tuberculosis, there were statistical differences (*p* < 0.05) in symmetry, homogeneity, border, fusion and CDFI pattern (dendritic type was more common in the sarcoidosis group). While comparing sarcoidosis and malignancy group, there were statistically significant variation in symmetry and CDFI pattern (other type was more common in the malignancy group and dendritic type was more common in the sarcoidosis group). While comparing sarcoidosis with the inflammatory group, there were statistically significant differences (*p* < 0.05) in symmetry, longitudinal meridian, lymphatic hilum, CDFI pattern (portal type was more common in the inflammatory group, and dendritic type was only observed in the sarcoidosis group) and elasticity score.

**Table 4:** Neck ultrasound (NUS) features in sarcoidosis versus non-sarcoidosis groups

Category	Sarcoidosis group	Control subjects							*p* value^4^
		Tuberculosis Group	*p* value^1^	Malignancy Group	*p* value^2^	Inflammatory Group	*p* value^3^	Total	
Symmetry									
Bilateral	18 (90.0%)	5 (21.7%)	< 0.001	10 (45.5%)	0.002	10 (43.5%)	0.001	25 (36.8%)	< 0.001
Unilateral	2 (10.0%)	18 (78.3%)		12 (54.5%)		13 (56.5%)		43 (63.2%)	
Number									
1	0 (0)	0 (0)	–	1 (4.5%)	1.000	5 (21.7%)	0.082	6 (8.8%)	0.383
> 1	20 (100%)	23 (100%)		21 (95.5%)		18 (78.3%)		62 (91.2%)	
Longitudinal meridian(mm)									
≥ 10	12 (60.0%)	19 (82.6%)	0.099	19 (86.4%)	0.052	3 (13.0%)	0.001	41 (60.3%)	0.981
< 10	8 (40.0%)	4 (17.4%)		3 (13.6%)		20 (87.0%)		27 (39.7%)	
Aspect ratio									
< 2	14 (70.0%)	19 (82.6%)	0.539	20 (90.9%)	0.361	13 (56.5%)	0.362	52 (76.5%)	0.557
≥ 2	6 (30.0%)	4 (17.4%)		2 (9.1%)		10 (43.5%)		16 (23.5%)	
Lymphatic hilum									
Presence	0 (0)	1 (4.3%)	1.000	0 (0)	–	16 (69.6%)	< 0.001	17 (25.0%)	0.030
Absence	20 (100%)	22 (95.7%)		22 (100%)		7 (30.4%)		51 (75.0%)	
Echo									
Hyperechoic	0 (0)	1 (4.3%)	1.000	0 (0)	–	0 (0)	–	1 (1.5%)	0.513
Hypoechoic	20 (100%)	22 (95.7%)		22 (100%)		23 (100%)		67 (98.5%)	
Homogeneity									
Uniform	15 (75.0%)	3 (13.0%)	< 0.001	12 (54.5%)	0.167	15 (65.2%)	0.486	30 (44.1%)	0.015
Non-uniform	5 (25.0%)	20 (87.0%)		10 (45.5%)		8 (34.8%)		38 (55.9%)	
Border									
Clear	20 (100%)	13 (56.5%)	0.003	21 (95.5%)	1.000	23 (100%)	–	57 (83.8%)	0.124
Unclear	0 (0)	10 (43.5%)		1 (4.5%)		0 (0)		11 (16.2%)	
Calcification									
Presence	0 (0)	4 (17.4%)	0.152	0 (0)	–	0 (0)	–	4 (5.9%)	0.617
Absence	20 (100%)	19 (82.6%)		22 (100%)		23 (100%)		64 (94.1%)	
Fusion									
Presence	0 (0)	9 (39.1%)	0.006	0 (0)	–	0 (0)	–	9 (13.2%)	0.194
Absence	20 (100%)	14 (60.9%)		22 (100%)		23 (100%)		59 (86.8%)	
CDFI pattern									
Portal	3 (15.0%)	2 (8.7%)	0.027	0 (0)	0.001	17 (73.9%)	< 0.001	19 (27.9%)	< 0.001
Dendritic	9 (45.0%)	2 (8.7%)		1 (4.5%)		0 (0)		3 (4.4%)	
Annular	1 (5.0%)	4 (17.4%)		0 (0)		0 (0)		4 (5.9%)	
Other	7 (35.0%)	15 (65.2%)		21 (95.5%)		6 (26.1%)		42 (61.8%)	
Elasticity score									
≥ 3	16 (80.0%)	14 (60.9%)	0.173	16 (72.7%)	0.849	6 (26.1%)	< 0.001	36 (52.9%)	0.030
< 3	4 (20.0%)	9 (39.1%)		6 (27.3%)		17 (73.9%)		32 (47.1%)	

CDFI: Color Doppler Flow Imaging; ^1^Sarcoidosis versus Tuberculosis Group; ^2^Sarcoidosis versus Malignancy Group; ^3^Sarcoidosis versus Inflammatory Group; ^4^Sarcoidosis versus Non-sarcoidosis groups

The distribution of sarcoidosis was discovered to be more bilateral when compared with non-sarcoidosis groups (18/20, 90.0% vs. 25/68, 36.8%, respectively). 100% of sarcoidosis lesion showed no lymphatic hilum, which was significantly different from inflammatory group (*p* < 0.05).

### Contrast-enhanced ultrasound (CEUS) features

**Fig. 1:**
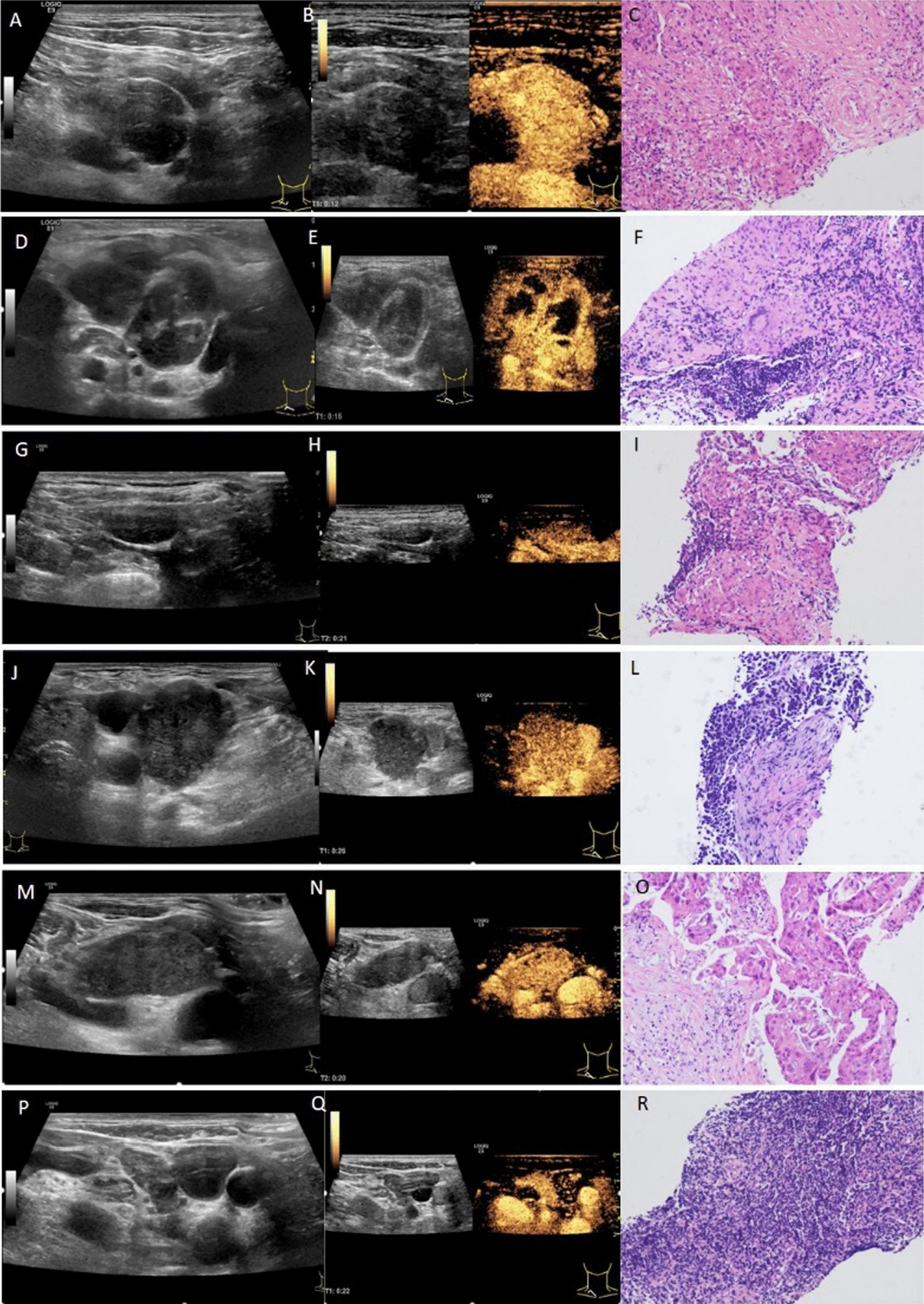
**A** NUS showed right supraclavicular lymphadenopathy, and the target lymph node was hypoechoic, uniform, no lymph hilum, clear border and no fusion. **B** CEUS showed that the target lymph node showed uniform and high enhancement and there was no non-enhanced area at the peak. **C** Pathological specimens obtained by ultrasound-guided percutaneous biopsy showed granuloma. (Case1, a 34-year-old male). **D** NUS showed right supraclavicular lymph node was enlarged, and the target lymph node was hypoechoic, non-uniform, no lymph hilum, clear border and no fusion. **E** CEUS showed that the target lymph node showed non-uniform and high enhancement, and there were large irregular areas without enhancement at the peak. **F** Pathological specimens obtained by ultrasound-guided percutaneous biopsy showed necrotizing granuloma. (Case2, a 22-year-old male). **G** NUS showed right supraclavicular lymph node was enlarged, and the target lymph node was hypoechoic, uniform, no lymphatic hilum, clear border and no fusion. **H** CEUS showed that the target lymph node showed uniform and equal enhancement, and there was no non-enhanced area at the peak. **I** Pathological specimens obtained by ultrasound-guided percutaneous biopsy showed granulomatous lesions, no necrosis, acid-fast found a small number of positive bacilli, considering the possibility of tuberculous lesions. (Case3, a 49-year-old female). **J** NUS showed right supraclavicular lymph node was enlarged, and the target lymph node was hypoechoic, uniform, no lymphatic hilum, clear border and no fusion. **K** CEUS showed that the target lymph node showed uniform and high enhancement, and there was no non-enhanced area at the peak. **L** Pathological specimens obtained by ultrasound-guided percutaneous biopsy showed metastatic non-small cell carcinoma, tending to adenocarcinoma. (Case4, a 65-year-old male). **M** NUS showed right supraclavicular lymph node was enlarged, and the target lymph node was hypoechoic, uniform, no lymphatic hilum, clear border and no fusion. **N** CEUS showed that the target lymph node showed non-uniform and high enhancement, and there were small irregular non-enhanced areas at the peak. **O** Pathological specimens obtained by ultrasound-guided percutaneous biopsy showed metastatic small cell carcinoma. (Case5, a 78-year-old male). **P** NUS showed right supraclavicular lymph node was enlarged, and the target lymph node was hypoechoic, non-uniform, lymphatic hilum, clear border and no fusion. **Q** CEUS showed that the target lymph node showed uniform and high enhancement, and there was no non-enhanced area at the peak. **R** Pathological specimens obtained by ultrasound-guided percutaneous biopsy showed lymphoid tissue hyperplasia (Case6, a 60-year-old male). Hematoxylin–eosin stain was used for histopathology and magnification × 100

There were statistically significant differences in the enhanced mode of ultrasound contrast agent, homogeneity, presence or absence of necrosis between the sarcoidosis and non-sarcoidosis with further grouping them in the tuberculosis or malignancy groups which is tabulated in Table 5, Fig. 1.

**Table.5:** Contrast-enhanced ultrasound (CEUS) features in sarcoidosis versus non-sarcoidosis groups

Category	Sarcoidosis group	Control subjects							*p* value^4^
		Tuberculosis group	*p* value^1^	Malignancy group	*p* value^2^	Inflammatory group	*p* value^3^	Total	
Enhanced mode									
Centripetal	0 (0)	13 (56.5%)	< 0.001	16 (72.7%)	< 0.001	4 (17.4%)	< 0.001	33 (48.5%)	< 0.001
Centrifugal	3 (15.0%)	6 (26.1%)		1 (4.5%)		19 (82.6%)		26 (38.2%)	
Polycentric	17 (85.0%)	2 (8.7%)		3 (13.6%)		0 (0)		5 (7.4%)	
Annular	0 (0)	2 (8.7%)		2 (9.1%)		0 (0)		4 (5.9%)	
Enhanced degree									
Low/equal	2 (10.0%)	2 (8.7%)	0.950	0 (0)	0.647	0 (0)	0.210	2 (2.9%)	0.471
High	18 (90.0%)	21 (91.3%)		22 (100%)		23 (100%)		66 (97.1%)	
Homogeneity									
Uniform	20 (100%)	4 (17.4%)	< 0.001	10 (45.5%)	< 0.001	23 (100%)	–	37 (54.4%)	< 0.001
Non-uniform	0 (0)	19 (82.6%)		12 (54.5%)		0 (0)		31 (45.6%)	
Necrosis									
Presence	0 (0)	19 (82.6%)	< 0.001	12 (54.5%)	< 0.001	0 (0)	–	31 (45.6%)	< 0.001
Absence	20 (100%)	4 (17.4%)		10 (45.5%)		23 (100%)		37 (54.4%)	
Arrival time (s)	10.4 ± 2.6	11.3 ± 3.1		13.3 ± 3.6		11.4 ± 3.0			
Peak time (s)	15.5 ± 3.7	17.8 ± 3.9		21.1 ± 5.1		17.7 ± 3.7			
Wash-out time (s)	22.3 ± 4.5	25.9 ± 4.0		28.4 ± 6.2		27.1 ± 3.2			

^1^Sarcoidosis versus Tuberculosis Group; ^2^Sarcoidosis versus Malignancy Group; ^3^Sarcoidosis versus Inflammatory Group; ^4^Sarcoidosis versus Non-sarcoidosis groups

**Fig. 2:**
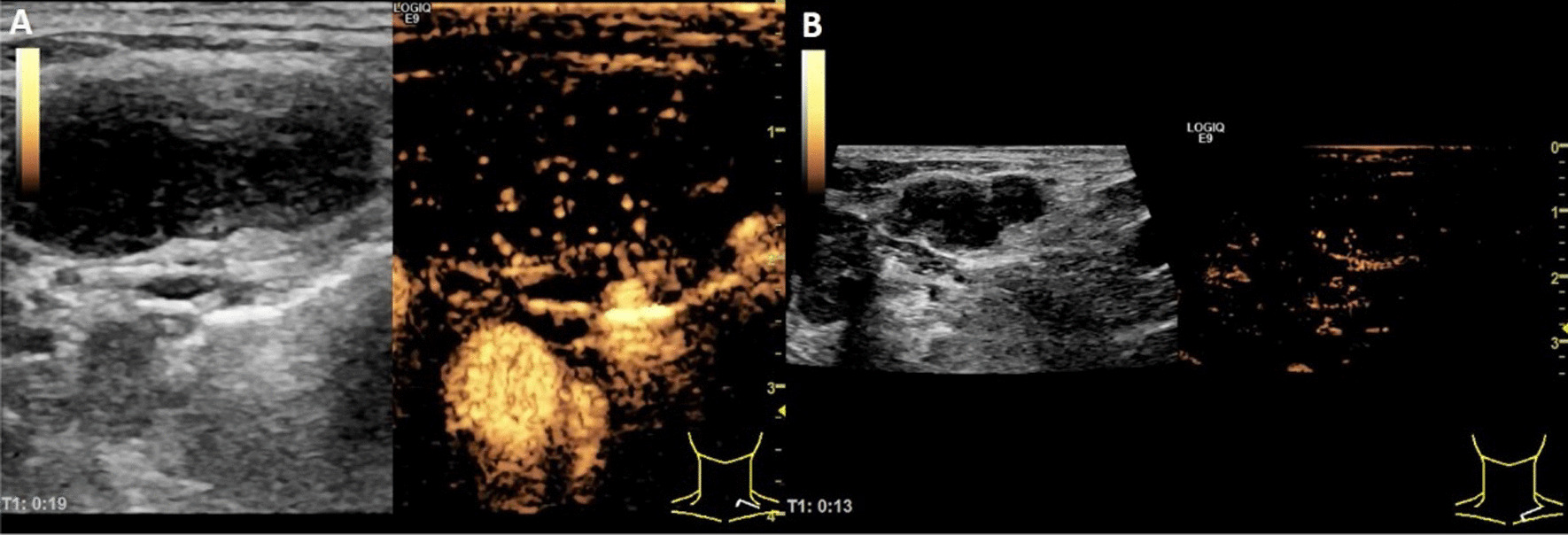
**A** CESU manifestation of sarcoidosis is that the enhancement pattern is polycentric. **B** The metastatic lymph node of lung cancer is centripetal

As to the enhanced mode of ultrasound contrast agent, the sarcoidosis group was mainly polycentric type (17/20, 85%) Fig. 2a, without centripetal or annular type. However, centripetal type (Fig. 2b) was more common in tuberculosis and malignancy groups (56.5%, 72.7%, respectively).

While considering the homogeneity, the perfusion in both sarcoidosis group and inflammation group were 100% uniform. While mainly the perfusion of tuberculosis lymph nodes were found to be non-uniform. Similarly, while regarding the necrosis, sarcoidosis lesions were 100% without any necrosis. 19/23 (82.6%) in tuberculosis and 12/22 (54.5%) in malignancy group showed necrosis.

### The diagnosis value of ultrasound features in sarcoidosis versus non-sarcoidosis

**Fig. 3:**
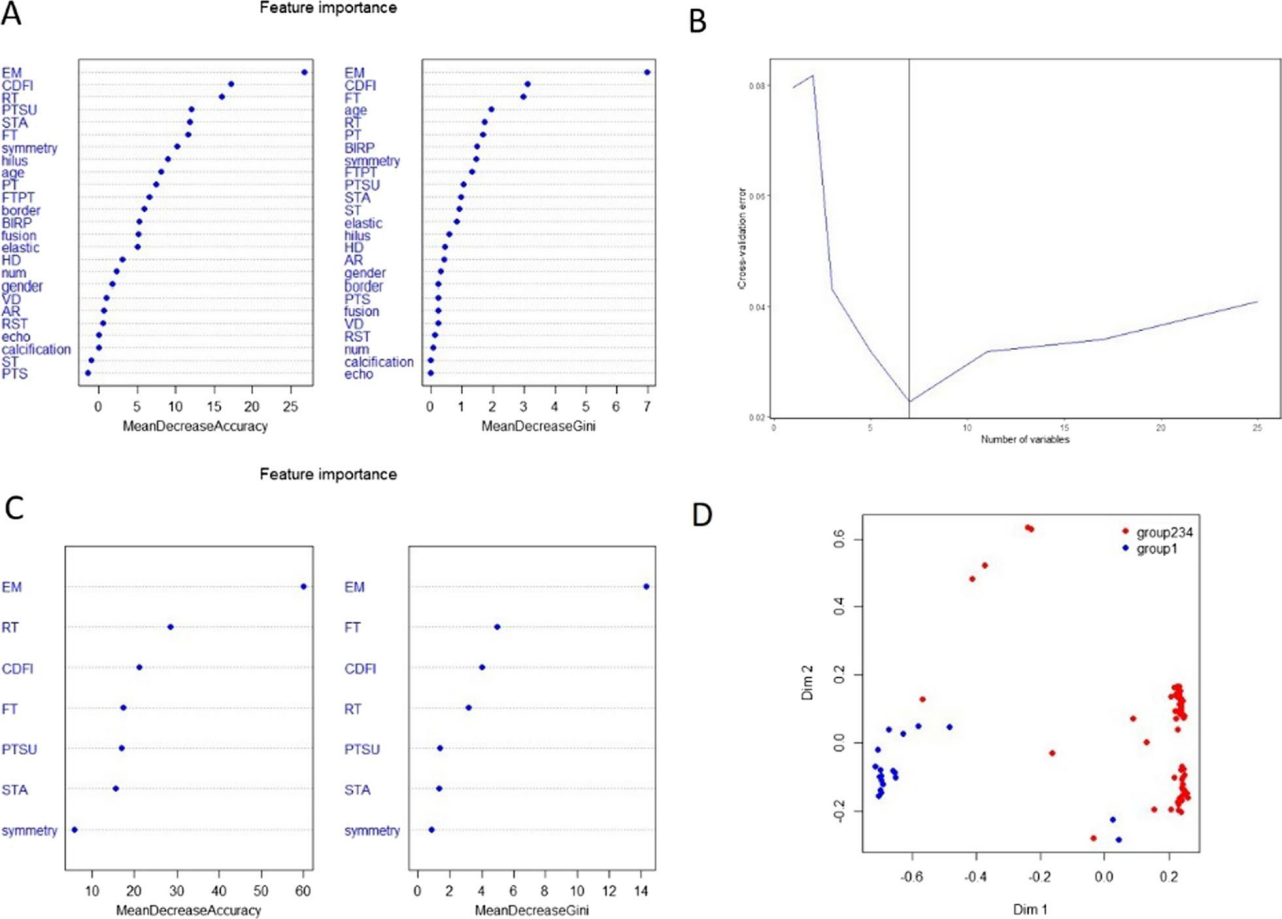
**A** 25 characteristics from NUS and CEUS were considered for diagnosis analysis. **B**, **C** Seven most significant characteristics were entered into the two classification analysis model. **D** The percentage correction of prediction was 90%

The seven characteristics were finally entered into the two classification analysis model which included enhancement mode (EM), resolution time (RT), CDFI, fading time (FT), peaking state-uniformity (PTSU), strengthen the area (STA) and symmetry. The percentage correction of prediction was 90% (18/20) (Fig. 3).

## Discussion

Sarcoidosis is a systemic disease in which intrathoracic lymph nodes, lungs and cervical lymph nodes are also commonly involved. The application value of neck ultrasound in sarcoidosis is worth exploring. Comparing with non-sarcoidosis groups, the characteristic ultrasound imaging features of sarcoidosis include: symmetry, non-existence of lymphatics, uniformity, and dendritic CDFI pattern are more common with elasticity score ≥ 3. The characteristic CEUS imaging features of sarcoidosis include: multi-center filling, uniform lesion and absence of necrosis.

Sarcoidosis can affect almost any organ. Bilateral hilar lymphadenopathy is its characteristic manifestation often accompanied by peripheral lymph node involvement. In our study, 90.0% (18/20) of bilateral cervical lymph nodes were symmetrically enlarged in the sarcoidosis group, and were comparable to non-sarcoidosis groups which included the tuberculosis group, tumor group, and inflammation group demonstrating highly significant statistical differences.

Normal lymphatic portal structure is formed by lymph nodes’ arteriovenous structure, fat and lymph sinus located in the central part of the lymph node. Schmid-Bindert G et al. considered that the disappearance of the lymphatic portal structure is one of the ultrasound features to identify benign and malignant lymph nodes [26]. However benign diseases such as tuberculosis and sarcoidosis of the lymph nodes can cause the destruction of the lymphatic structure. Dhorria et al. conducted a retrospective analysis of 165 patients with lymph node tuberculosis and sarcoidosis and found that the proportion of lymphatic structure involved in sarcoidosis was 13.8% and 11.4% in lymph node tuberculosis [27]. Jakubowski et al. found that sarcoidosis lymph nodes lack the lymphatic hilum or have no clearly visible lymphatic hilum [28]. Wang and colleagues found that only 2 out of 193 cases of sarcoidosis lymph nodes had lymphatic hilum, while 11 out of 37 cases of nodular tuberculosis had lymphatic hilum, hence, considered that the absence of lymphatic hilum is an important feature of sarcoidosis [29]. However, some papers indicate the presence of lymphatic hilum is characteristic. Hasegawa et al. found that in 42 lymph nodes, the lymphatic hilum could be observed in 71.4% of cases [30]. In addition, Dincer et.al. found that the presence of lymphatic hilum may be distinctive echoic features of lymph nodes with sarcoidosis [31]. The reasons for these differences may be as follows: (1) Cervical lymph node ultrasonography is affected by the patient's cervical mobility, respiratory rate, ultrasonic penetration of tissue and other factors. (2) In this study, there were only 20 cases of sarcoidosis lymph nodes, the sample size was small, and there may be selection bias.

In our study, there were 20 cases of sarcoidosis and NUS did not find lymphatic hilum which was significantly different from non-sarcoidosis group. Lymph node enlargement in the inflammation group was reactive hyperplasia and the structure of the lymphatic gate was not obliterated. Theoretically, structure of the lymphatic hilum should be intact in all the cases, but in 30.4% (7/23) of the cases, lymph nodes in the inflammation group of this study lymphatic hilum was not visible which is consistent with previous studies. According to Dudau et al., the reactive hyperplasia of lymph nodes in the cancer drainage area can be easily confused with metastatic lymph nodes [22].

In terms of ultrasound contrast agent filling methods, our results showed that the sarcoidosis group was mainly multicenter (85.0%, 17/20), and the tuberculosis group and tumor group were mainly centripetal (56.5% v.s. 72.7%, respectively) whereas the inflammation group was mainly eccentric (82.6%, 19/23). Histologically, sarcoidosis was characterized by non-caseating granuloma. Within the affected lymph nodes, the usual structure was obscured by multiple distinct, "tight" and discrete granulomas [32]. The typical manifestation of granuloma was concentric rings. Therefore, when the target lymph node of the sarcoidosis group was undergoing contrast-enhanced ultrasound examination, the contrast agent was filled in a multi-center manner. In the tuberculosis group, because of necrosis or destruction of the lymphatic portal in the lymph node, and granulomatous inflammatory reaction in the surrounding area, it appeared as centripetal type mode. Lymph nodes in the tumor group invaded the cortex under the lymph node capsule, causing tumor angiogenesis and lymphatic hilar vessels to shift or even disappear. The new blood vessels were mainly distributed in the marginal area of the lymph nodes, and the branches were distributed centripetally resulting in metastatic lymph nodes. For centripetal filling, the lymph nodes in the inflammatory group were eccentric due to the presence of the lymphatic hilum. The contrast agent first entered the lymph nodes from the arteries and veins of the lymph hilum then spread to the entire lymph nodes [33].

In terms of the uniformity of enhancement, both the sarcoidosis group and the inflammatory nodules group showed uniform enhancement, while the tuberculosis group and the tumor group showed an uneven enhancement. The reason might be because the tuberculosis group is prone to have caseous necrosis of the lymph nodes. Likewise, for tumor group, tumor cells block the blood vessels or hypercoagulable blood leads to thrombosis.

Necrosis is more common in tuberculosis and tumors. In general, sarcoidosis is not a condition to consider necrosis initially as most sarcoid granulomas are not accompanied by necrosis. Although there are individual sarcoidosis-like necrotizing granulomas reported in the previous literature. Ultrasound contrast agent is a blood pool imaging agent, which can accurately reflect the microcirculation blood perfusion inside the lesion and help distinguish the necrotic area within the lesion [34].

This study preliminarily explored the application of NUS and CEUS in the diagnosis of sarcoidosis. They can reflect partial vascularization forming a different impression of pseudo-ischemic area which can more truly reflect the active ingredients in the lesion. In our study, 20 target lymph nodes in the sarcoidosis group showed uniform enhancement during CEUS and no non-enhanced area indicated that there was no necrosis. Therefore, necrosis found through CEUS can be used to rule out sarcoidosis for differential diagnosis.

This study has the following limitations: (1) this study is a retrospective study of a single center and it is difficult to avoid the small number of cases and the deviation of the disease, (2) the prospective verification has not been performed to evaluate its diagnostic efficacy, (3) the authors didn’t combine the quantitative parameters obtained by CEUS time-intensity curve analysis to obtain more valuable diagnostic results. In future, a more reasonable multicenter prospective cohort study can be established to further verify the value of NUS combined with CEUS in the diagnosis of patients with sarcoidosis involving cervical lymph nodes to obtain a better result.

## Conclusion

In summary, sarcoid lymph nodes displayed under conventional ultrasound and CEUS have certain specific characteristics which are helpful for the diagnosis and differential diagnosis of sarcoidosis and have a great potential to become an important non-invasive complement to existing diagnostic methods. The ultrasound diagnosis model established by the binary classification algorithm helps to distinguish the diagnosis of sarcoidosis from other granulomatous non-sarcoidosis lesions such as malignancy, tuberculosis and inflammatory lymph nodes.

## Data Availability

All data regarding the included participants during the study are available from the corresponding author by email request.

## Abbreviations

NUS: Neck ultrasound
CEUS: Contrast-enhanced ultrasound
FNA: Fine-needle aspiration
CNB: Core needle biopsy
EM: Enhanced mode
RT: Resolution time
CDFI: Color Doppler flow imaging
FT: Fading time
PTSU: Peaking state-uniformity
STA: Strengthen the area
LNS: Location, number and size
TB: Tuberculosis
SD: Standard deviation
ANOVA: Analysis of variance
EBUS-TBNA: Endobronchialultrasound-guidedtransbronchialneedleaspiration
EBB: Endobronchialbiopsy
TBLB: Transbronchiallungbiopsy
